# Supplementary material for: Analysis of erythrocyte signalling pathways during Plasmodium falciparum infection identifies targets for host-directed antimalarial intervention
Source: Nat Commun. 2020 Aug 11;11:4015. doi: 10.1038/s41467-020-17829-7 (PMC7419518; doi:10.1038/s41467-020-17829-7)

Analysis of erythrocyte signalling pathways during *Plasmodium falciparum* infection identifies targets for host-directed antimalarial intervention

Adderley *et al*.

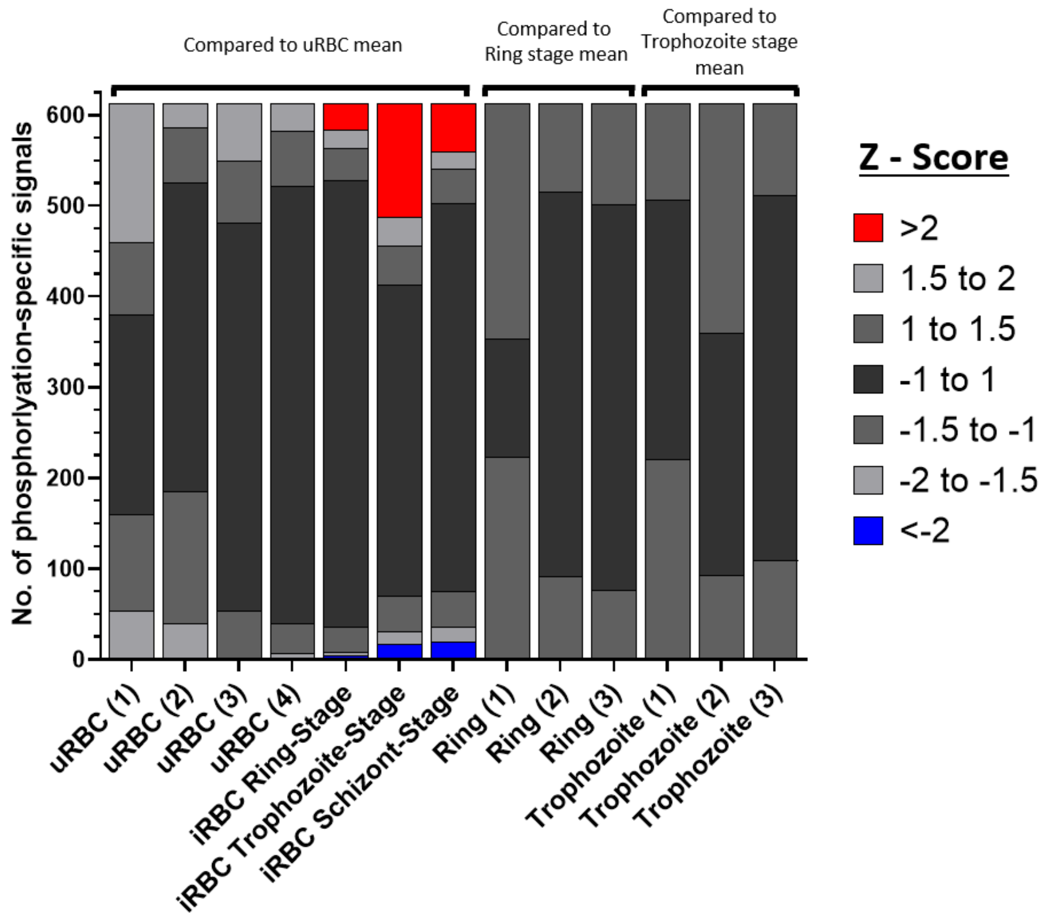

Supplementary Figure 1 – Distribution of Z-scores for each sample. All microarray signals from each sample (four from uRBCs and the mean signal for each iRBC harboring parasites at the ring (n=3), trophozoite (n=3) and schizont stage (n=2)) were compared to the uninfected erythrocyte mean for each signal and internally for each of the iRBC stages, and the Z-score determined for each phosphorylation-specific signal. The Z-score is defined as the number of standard deviations from the uRBC mean for each sample; a positive or negative value indicates the direction of the signal (increase or decrease, respectively) from the uRBC, or iRBC stage mean. No signals from the uRBC samples (n=4) had associated Z-scores above 2 or below -2, while the three iRBC time points showed clear variations from uRBCs. Internally the iRBC samples also showed low variation as there were no Z-scores above 2 or below -2 from the iRBC stage mean. Schizont stage Z-scores not determined as n=2.

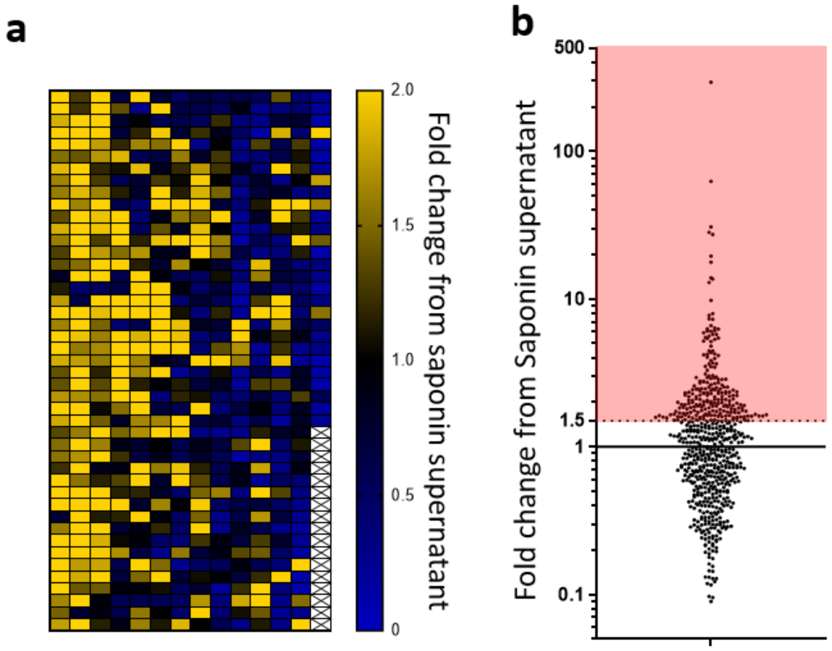

Supplementary Figure 2 – Summary of the saponin lysis comparison array. a) heatmap of the 613 phosphorylation-specific signals on the antibody microarray. Each pixel represents a single antibody, were the colour represents the fold change difference between the saponin supernatant (erythrocyte cytoplasm) and the saponin pellet (parasite material). Yellow pixels indicate that the signal was stronger in the saponin pellet material, indicating likely cross-reactivity of the corresponding antibody. **b)** dot plot of the phosphorylation-specific signals on the saponin lysis comparison array, the red region indicates the signals removed due to potential cross-reactivity (signals above a fold change of 1.5)

		uRBC	Ring stage		Trophozoite stage		Schizont stage	
**Protein**	**Phospho Site (Human)**	**Error (%)**	**Fold change**	**Error (%)**	**Fold change**	**Error (%)**	**Fold change**	**Error (%)**
PKCd	Y313	18	0.89	11	0.86	6	1.46	2
	Y313	6	0.90	12	0.82	10	1.37	7
	Y313	5	0.88	6	1.19	17	1.20	12
	S645	8	0.79	18	1.11	10	1.17	11
	S664	28	0.68	16	0.55	65	0.93	26
	T507	9	1.05	12	1.96*	9	2.05*	20
	Pan-specific	16	0.91	15	1.59	14	1.53*	13
PKCq	S676	25	0.73	8	0.87	8	0.80	15
	S695	30	1.00	23	1.90*	29	1.43*	21
	S695	15	0.86	13	1.59*	5	1.49	12
	Y545	5	1.08	6	1.80*	11	1.47	3

* $p<0.05$

Supplementary Figure 3 - Summary of the antibody microarray signals for the PKC isoforms delta and theta, that were not flagged as cross-reactivity or low signal intensity. Fold change indicates the fold change from the uRBC control, yellow = increase, blue = decrease. Asterisk indicates which of the fold changes were noted as significant changes ($p<0.05$, unpair two-tailed T-Test).

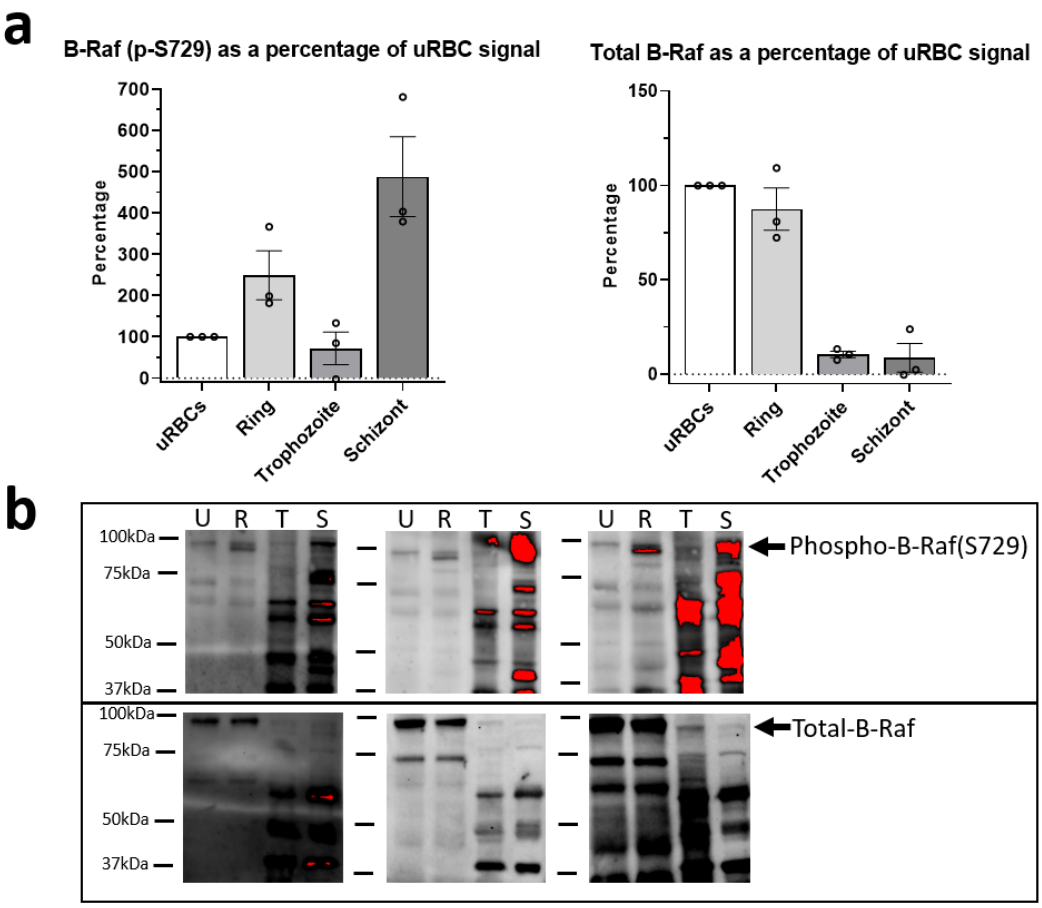

Supplementary Figure 4 – Complete Western blots for total B-Raf and Phospho-B-Raf(S729) signal across 3 replicates. **a)** Summary bar graph illustrating the densitometry results for Phospho-B-Raf signal (left) and total B-Raf signal (right) on Western blots illustrated in panel b (n=3 independent experiments). Results represent the signal as a percentage of the uninfected (uRBC) control. Error bars represent the standard error of the mean. **b)** Western blot results of three replicates using both total B-Raf and pan-B-Raf antibodies. A phospho-specific antibody to B-Raf (S729) detected phosphorylated B-Raf in ring-stage infected erythrocytes (upper panel). A pan-B-Raf antibody detected B-Raf in the uninfected and ring-stage infected erythrocyte samples, with a strong decrease at the trophozoite and schizont stages (bottom panel). Black arrow indicates the expected size of B-Raf. Overexposed blots are presented here (red sections in the images) to facilitate easier viewing of fainter bands.

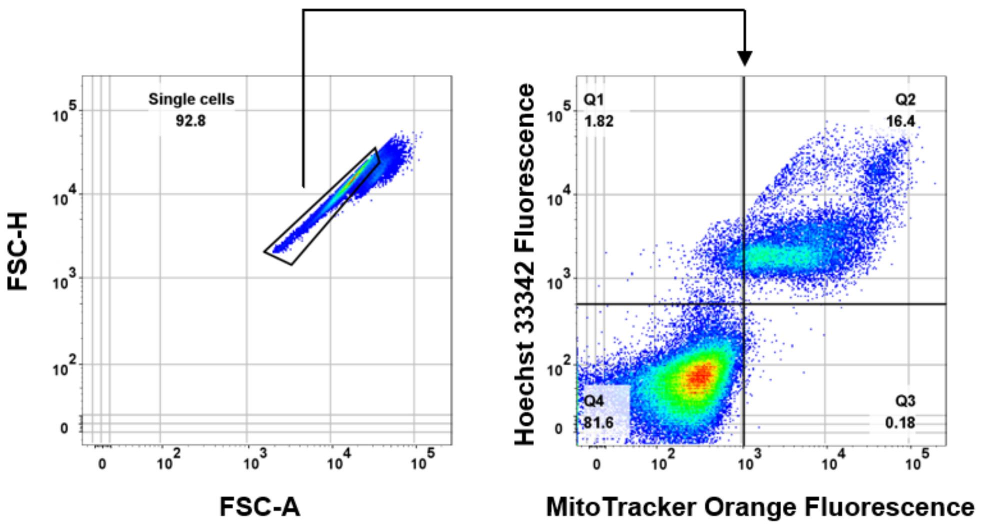

Supplementary Figure 5 – Flow cytometry gating strategy used to determine live *P. falciparum* parasitemia following inhibitor treatment. Gating for single cells (left), gating for live parasitised cells (right), Q1 represents dead *P. falciparum* parasites, Q2 live parasites, Q4 uninfected erythrocytes, Percentage of each quadrant is listed under the quadrant label. Hoeschst-33342 fluorescence measured using a UV-379 laser (filter 450/50), MitoTracker Orange fluorescence measured using YG-585 (filter (585/15). Staining for flow cytometry analysis used 2µM Hoechst-33342 staining (8 min) and 75nM MitoTracker Orange staining (25 min); 100,000 events were recorded using a LSR BDFortessaTM and analysed by FlowJo software (Tree Star).

Supplementary Figure 6 - Uncropped original Western blot scans

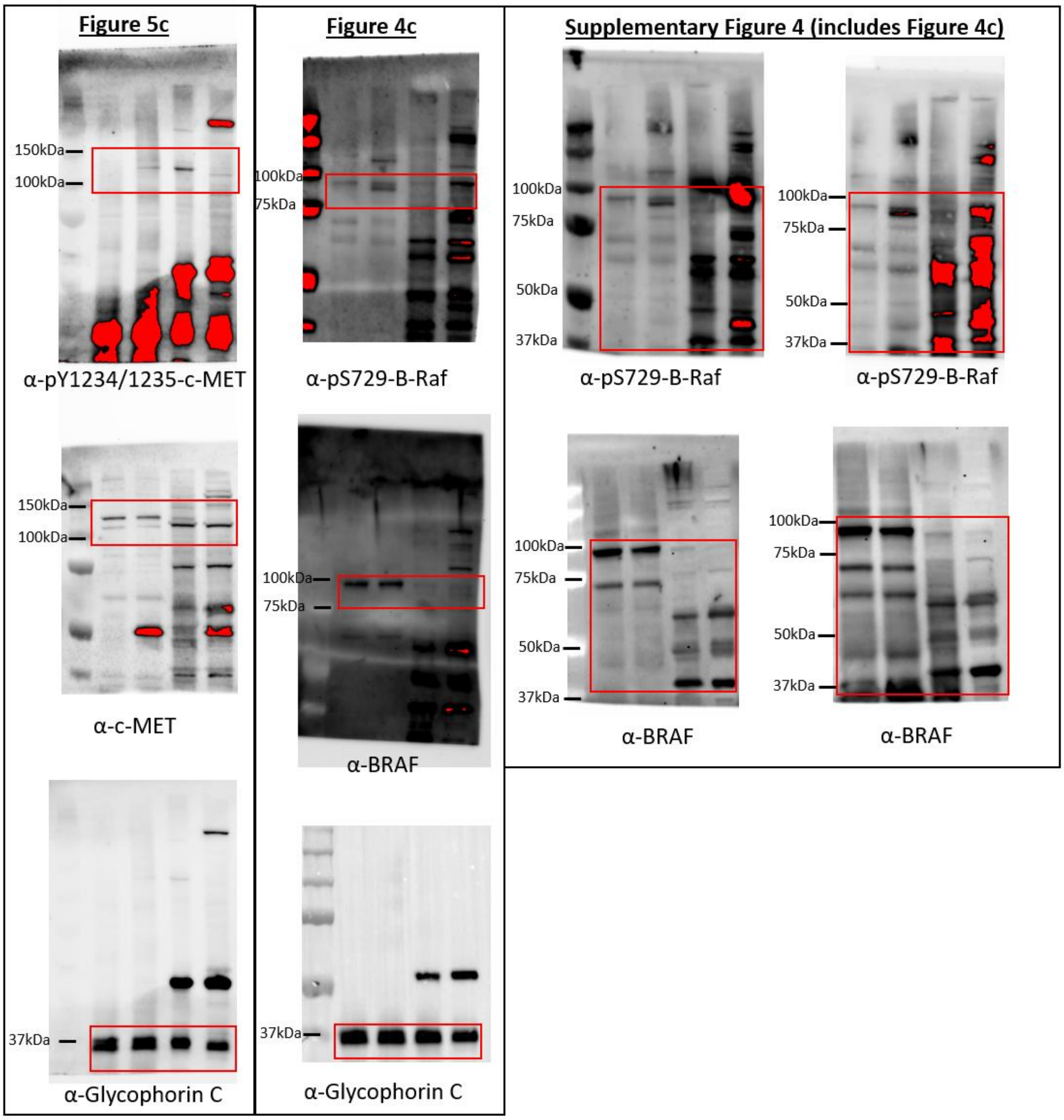

Supplement: Supplementary file 1 — Supplementary Information [file 41467_2020_17829_MOESM1_ESM.pdf]
