# Supplementary material for: Analysis of erythrocyte signalling pathways during Plasmodium falciparum infection identifies targets for host-directed antimalarial intervention
Source: Nat Commun. 2020 Aug 11;11:4015. doi: 10.1038/s41467-020-17829-7 (PMC7419518; doi:10.1038/s41467-020-17829-7)
Supplement: Supplementary file 3 — Reporting Summary [file 41467_2020_17829_MOESM3_ESM.pdf]

nature research

Corresponding author(s): Prof Christian Doerig

Last updated by author(s): Jul 7, 2020

Reporting Summary

Nature Research wishes to improve the reproducibility of the work that we publish. This form provides structure for consistency and transparency in reporting. For further information on Nature Research policies, see our Editorial Policies and the Editorial Policy Checklist.

Statistics

For all statistical analyses, confirm that the following items are present in the figure legend, table legend, main text, or Methods section.

n/a	Confirmed	
[ ]	[x]	The exact sample size (*n*) for each experimental group/condition, given as a discrete number and unit of measurement
[ ]	[x]	A statement on whether measurements were taken from distinct samples or whether the same sample was measured repeatedly
[ ]	[x]	The statistical test(s) used AND whether they are one- or two-sided<br>*Only common tests should be described solely by name; describe more complex techniques in the Methods section.*
[ ]	[x]	A description of all covariates tested
[ ]	[x]	A description of any assumptions or corrections, such as tests of normality and adjustment for multiple comparisons
[ ]	[x]	A full description of the statistical parameters including central tendency (e.g. means) or other basic estimates (e.g. regression coefficient) AND variation (e.g. standard deviation) or associated estimates of uncertainty (e.g. confidence intervals)
[ ]	[x]	For null hypothesis testing, the test statistic (e.g. *F*, *t*, *r*) with confidence intervals, effect sizes, degrees of freedom and *P* value noted<br>*Give P values as exact values whenever suitable.*
[x]	[ ]	For Bayesian analysis, information on the choice of priors and Markov chain Monte Carlo settings
[x]	[ ]	For hierarchical and complex designs, identification of the appropriate level for tests and full reporting of outcomes
[x]	[ ]	Estimates of effect sizes (e.g. Cohen's *d*, Pearson's *r*), indicating how they were calculated

Our web collection on statistics for biologists contains articles on many of the points above.

Software and code

Policy information about availability of computer code

Data collection	I-Control V1.10.4.0, BD FACSDiva V9.0
Data analysis	GraphPad PRISM V8.2.1, FlowJo V10.6.2, Image Lab V6.0.1

For manuscripts utilizing custom algorithms or software that are central to the research but not yet described in published literature, software must be made available to editors and reviewers. We strongly encourage code deposition in a community repository (e.g. GitHub). See the Nature Research guidelines for submitting code & software for further information.

Data

Policy information about availability of data

All manuscripts must include a data availability statement. This statement should provide the following information, where applicable:

- Accession codes, unique identifiers, or web links for publicly available datasets
- A list of figures that have associated raw data
- A description of any restrictions on data availability

All raw data pertaining to this study are available in Supplementary Table 1 to 3.

Field-specific reporting

Life sciences study design

All studies must disclose on these points even when the disclosure is negative.

Sample size	Similar studies have been conducted with a single microarray datasets (Hasqshenas et al. Nat. Commun. 2017 & Hasqshenas et al. Cell Rep. 2019), and this was deemed acceptable by editorial boards of high profile journals. Here we established robustness through multiple arrays n=2 (Schizont time point) n=3 (Ring and Trophozite time points), each microarray contains technical replicates.
Data exclusions	The complete datasets are available for each of the antibody microarray experiments. These datasets were filtered for cross-reactivity and low-intensity signals for all of the analysis conducted in this paper. The cross-reactivity exclusion criteria was not -preestablished.
Replication	3 biological replicates for the P. falciparum ring and trophozoite samples, 2 biological replicates for the P. falciparum schizont sample were assessed by antibody micorarray. Each array contains two spots for each antibody, providing 2 technical replicates. All attempts at replication were successful. Western blots using c-MET antibodies were replicated twice, Western blot using B-Raf antibodies were replicated three times, all attempts at Western blot data replication was successful. Kinase inhibitor assay were between 2-5 times, each experiment states the number of replicates complete in the figure legend, with all attempts at replication being successful.
Randomization	The study design is based on the comparison of infected vs non-infected cells at different time-points and did not included randomization. All signaling changes were statistically assessed as stated in the manuscript.
Blinding	The study design is based on the comparison of infected vs non-infected cells at different time-points and did not included randomization. All signaling changes were statistically assessed as stated in the manuscript.

Reporting for specific materials, systems and methods

We require information from authors about some types of materials, experimental systems and methods used in many studies. Here, indicate whether each material, system or method listed is relevant to your study. If you are not sure if a list item applies to your research, read the appropriate section before selecting a response.

Materials & experimental systems

n/a	Involved in the study
☐	☒ Antibodies
☐	☒ Eukaryotic cell lines
☒	☐ Palaeontology and archaeology
☐	☒ Animals and other organisms
☒	☐ Human research participants
☒	☐ Clinical data
☒	☐ Dual use research of concern

Methods

n/a	Involved in the study
☒	☐ ChIP-seq
☐	☒ Flow cytometry
☒	☐ MRI-based neuroimaging

Antibodies

Antibodies used	Antibody micorarray (Kinexus Cat no. KAM900P), B-Raf (Polyclonal, Cat No.AB-PK535, Kinexus), c-MET (Clone:D1C2, cat no.8198, Cell Signaling Technologies), B-Raf (Clone:OTI4B2, Cat no. OTI4B2, Bio-Rad), c-MET (Clone:D26, Cat No. 3077S, Cell Signaling Technologies), Glycophorin-C (Polyclonal, Cat no.ab175257, Abcam) Mouse Secondary-HRP (Clone: X1, Monash Antibody facility, Victoria Australia), Rabbit Secondary-HRP(Clone: D10, Monash Antibody facility, Victoria, Australia)
Validation	Kinexus antibody microarray – Manufacturer's website " highly validated antibody probes" " The printing of individual antibodies on our microarrays is validated by probing with dye-labeled anti-rabbit, anti-mouse and anti-goat secondary antibodies. Each microarray also has loading and antibody controls to ensure the amount of deposited protein is consistent on all fields.<br><br>B-Raf (Polyclonal, Cat No.AB-PK535, Kinexus) - From Kinexus website: " Strong immunoreactivity with immunogen peptide on dot blots. Very strong immunoreactivity with recombinant human B-Raf on protein dot blots."<br><br>B-Raf (Clone:OTI4B2, Cat no. OTI4B2, Bio-Rad) - From Bio-Rad website: 'Bio-Rad conducts rigorous in house testing to guarantee that all of our antibodies meet our internal benchmarks and perform in their designated applications as expected.'<br><br>c-MET (Clone:D1C2, cat no.8198, Cell Signaling Technologies) - From CST website: 'we adhere to the Hallmarks of Antibody Validation™, six complementary strategies that can be used to determine the functionality, specificity, and sensitivity of an antibody in any given assay. CST adapted the work by Uhlen, et. al., ("A Proposal for Validation of Antibodies." Nature Methods (2016)) to build the Hallmarks of Antibody Validation, based on our decades of experience as an antibody manufacturer.'<br><br>c-MET (Clone:D26, Cat No. 3077S, Cell Signaling Technologies) - From CST Website: 'we adhere to the Hallmarks of Antibody Validation™, six complementary strategies that can be used to determine the functionality, specificity, and sensitivity of an antibody in any given assay. CST adapted the work by Uhlen, et. al., ("A Proposal for Validation of Antibodies." Nature Methods (2016)) to build the Hallmarks of Antibody Validation, based on our decades of experience as an antibody manufacturer.'

Glycophorin-C (Polyclonal, Cat no.ab175257, Abcam) - From Abcam website: 'Antibodies are validated in western blot using lysates from cells or tissues that we have identified to express the protein of interest. Once we have determined the right lysates to use, western blots are run and the band size is checked for the expected molecular weight.'

Eukaryotic cell lines

Policy information about cell lines

Cell line source(s)	Plasmodium clone 3D7 was originally provided by the group who performed the cloning (Walliker group University of Edinburgh), and has been used in the Doerig lab ever since.
Authentication	Many genes have been cloned and sequenced over the years, all of which were consistent with 3D7 identity.
Mycoplasma contamination	The line was tested for Mycoplasma contamination prior to experiments described here, but not during the course of the experiments described in the paper, which took place over a duration of about three years.
Commonly misidentified lines (See ICLAC register)	no commonly misidentified cell lines were used in the study.

Animals and other organisms

Policy information about studies involving animals; ARRIVE guidelines recommended for reporting animal research

Laboratory animals	Mice were purchased from Envigo, kept in specific-pathogen-free conditions and subjected to regular pathogen monitoring by sentinel screening. They were housed in individually ventilated cages furnished with autoclaved aspen woodchip, fun tunnel and Nestlets at 21°C +/- 2°C under a 12:12 hr light-dark cycle at a relative humidity of 55 +/- 10%. They were fed a commercially prepared autoclaved dry rodent diet and water, both available ad libitum. The health of animals was monitored by routine daily visual health checks. Cohorts of seven female, 10-week-old BALB/c mice per treatment group were infected by i.v. inoculation with 106 infected erythrocytes. Assays to determine parasite growth were performed as described 76, using the reporter parasite line PbGFPLuccon (RMgm-29 in the rodent malaria genetic modification database, http://www.pberghei.eu/index.php?rmgm=29), which expresses a GFP-firefly luciferase fusion protein under the control of the constitutive eef1a promoter 77
Wild animals	no wild animals were used in the study.
Field-collected samples	no field collected samples were used in the study.
Ethics oversight	Animal research was conducted under licenses from the UK Home Office, and protocols were approved by the Animal Welfare and Ethical Review Body of the Wellcome Sanger Institute.

Note that full information on the approval of the study protocol must also be provided in the manuscript.

Flow Cytometry

Plots

Confirm that:

- [x] The axis labels state the marker and fluorochrome used (e.g. CD4-FITC).
- [x] The axis scales are clearly visible. Include numbers along axes only for bottom left plot of group (a 'group' is an analysis of identical markers).
- [x] All plots are contour plots with outliers or pseudocolor plots.
- [x] A numerical value for number of cells or percentage (with statistics) is provided.

Methodology

Sample preparation	Highly synchronous cultures of P. falciparum infected erythrocytes (0 - 4 hrs post-invasion) at 2% haematocrit were monitored at 12-hr time intervals over two full asexual intra-erythrocytic cycles (84 hrs) by flow cytometry. Live parasitaemia was quantitated by a dual-colour flow cytometry staining protocol using 2 µM Hoechst-33342 staining for 8 minutes and 75 nM MitoTracker Orange for 25 minutes. Staining was completed in v-bottom plates with two washes in complete RPMI. Stained cells were transferred to polypropylene tubes and diluted one in four in complete RPMI.<br>The Uninfected human erythrocytes were collected from the Australian Red Cross.
Instrument	LSR BDFortessaTM
Software	FlowJo software (Tree Star)
Cell population abundance	The abundance of infected and uninfected cells samples varied across the experiment as the cells were actively growing/ dying due to inhibitor treatment. Infected cell population varied from 0-10% of the total erythrocyte population. The relative abundance of infected vs uninfected cells was determined through UV-379 fluorescence (Hoechest 33342 staining).
Gating strategy	The gating strategy applied a quadrant grid between the Hoechst-33342 and Mitotracker Orange fluorescence. Positive Hoechst-33342 was defined as having a fluorescence intensity above 500 units. Positive Mitotracker Orange was defined as

having a fluorescence intensity above 700 units.

☒ Tick this box to confirm that a figure exemplifying the gating strategy is provided in the Supplementary Information.
