# Supplementary material for: Analysis of erythrocyte signalling pathways during Plasmodium falciparum infection identifies targets for host-directed antimalarial intervention
Source: Nat Commun. 2020 Aug 11;11:4015. doi: 10.1038/s41467-020-17829-7 (PMC7419518; doi:10.1038/s41467-020-17829-7)
Supplement: Supplementary file 4 — Description of Additional Supplementary Files [file 41467_2020_17829_MOESM4_ESM.pdf]

Description of Additional Supplementary Files

File Name: Supplementary Data 1
Description: Complete Kinexus antibody microarray raw data for all replicates. Raw data displays from left to right:

- the 4 uninfected red blood cell samples with supporting metrics,
- ring-stage P. falciparum-infected red blood cell signals with supporting metrics,
- trophozoite stage P. falciparum-infected red blood cell signals with supporting metrics,
- schizont-stage P. falciparum-infected red blood cell signals with supporting metrics
- two-tailed t-tests (three column, one per development stage ring, trophozoite and schizont)which compare each signal mean signal for the infected samples against the mean for the uninfected red blood cells equivalent signal.

File Name: Supplementary Data 2
Description: Complete Kinexus antibody microarray raw data for the array performed on saponin lysed late-stage P. falciparum-infected cells. The fold change value listed for each signal represents the fold change from the saponin supernatant and was used to define cross-reactive antibodies on the array.

File Name: Supplementary Data 3
Description: Complete Kinexus antibody microarray data from infected cells determined to be significantly different (two-tailed t-test, See Supplementary Data 1) from the uninfected red blood cell sample. Each of the three parasite stages (ring, trophozoite and schizont) is listed separately, with the respective fold change and p-values.
