# Supplementary material for: Analysis of erythrocyte signalling pathways during Plasmodium falciparum infection identifies targets for host-directed antimalarial intervention
Source: Nat Commun. 2020 Aug 11;11:4015. doi: 10.1038/s41467-020-17829-7 (PMC7419518; doi:10.1038/s41467-020-17829-7)
Supplement: Supplementary file 2 — Peer Review File [file 41467_2020_17829_MOESM2_ESM.pdf]

Editorial Note: This manuscript has been previously reviewed at another journal that is not operating a transparent peer review scheme. This document only contains reviewer comments and rebuttal letters for versions considered at *Nature Communications*.

Reviewers' Comments:

Reviewer #1:
Remarks to the Author:
All concerns were addressed fully. I have no more concerns nd support this manuscript for publication.

Reviewer #2:
Remarks to the Author:
I am happy that the authors have fully addressed the issues that I raised previously.
David Baker
